# Supplementary material for: Epidemiology and antimicrobial resistance of Enterobacteriaceae causing community-acquired urinary tract infections in Tétouan, Morocco (2022–2023)
Source: PLoS One. 2026 Apr 28;21(4):e0347958. doi: 10.1371/journal.pone.0347958 (PMC13124006; doi:10.1371/journal.pone.0347958)
Supplement: Attachment — Submitted filename: PONE-D-25-62063.docx [file pone.0347958.s002.docx]

Title of the manuscript: Epidemiology and Antimicrobial Resistance of Enterobacteriaceae Causing Community-Acquired Urinary Tract Infections in Tétouan, Morocco (2022–2023)

Review: December 11, 2025

Farouk et al. described the antimicrobial resistance of bacteria of Enterobacteriaceae family causing urinary tract infections from the hospital/medical laboratories in Tetouan, Morocco. Only two demographic variables gender and age were used to describe antibiotic resistance in bacteria. Further, very limited epidemiological analysis has been done. Many results presented in tables and figures are redundant. The manuscript has limited scope and could be of interest of limited readers.

The following are the comments on the manuscript.

1. The short title looks like the key words without any correct meaning. Please correct it.

2. Although prospective descriptive study has been mentioned in the design, there is no analysis of data as per prospective design. The prospective design has a core aspect of the analysis to track how the measured variables change or evolve over the defined time frame. But there is no such analysis in the manuscript to monitor the trends.

3. ESBL *E. coli* were 72.7%, which was wrongly calculated. It should be calculated using ESBL *E. coli* as numerator and total *E. coli* tested as denominator.

4. The resistance trends according to age and gender of the patient have not been discussed. I think these variables although by chance found associated in the results but are not correct variables for comparison.

5. Page 3, line 70, please italicize *Klebsiella pneumoniae*.

6. Page 4, line 104, the prospective design is not appropriate word since there is no such analysis supporting prospective design.

7. Nitrofurantoin is the most commonly used antibiotic for the antibiotic susceptibility test of urine isolates. But it has not been found to be used in this study.

8. The distribution of bacterial species according to gender and age has been presented which is not relevant. Similar is the case for distribution of resistance patterns by gender and age. It cannot be explained that pediatric isolates were more likely to harbor resistance to 225 cephalosporins and certain aminoglycosides compared with adult and geriatric isolates.

9. Table 1 and 2 have presentation of same data. However, there is difference in some data between two tables such as isolates in age group 18-24, 45-64, 65-79 and 80 or more.

10. Table 3 and figure 1, and table 4 and figure 2, present the same data. Therefore, one is redundant.

11. As stated by authors, there are several limitations of the study. There are very limited data described based on very few variables.
