# Supplementary material for: Epidemiology and antimicrobial resistance of Enterobacteriaceae causing community-acquired urinary tract infections in Tétouan, Morocco (2022–2023)
Source: PLoS One. 2026 Apr 28;21(4):e0347958. doi: 10.1371/journal.pone.0347958 (PMC13124006; doi:10.1371/journal.pone.0347958)
Supplement: Attachment — Submitted filename: Response to Editor and Reviewers.docx [file pone.0347958.s004.docx]

**Corresponding author: Ayoub Ez-Zari (PhD)**
Abdelmalek Essaâdi University,

Faculty of Science of Tetouan, Department of Biology,

Laboratory of Biology and Health, Food Science and Health

Research Team (UAE/U16FS),
BP 2121, 93002, Tetouan, Morocco
ayoub.ezz1990@gmail.com
27-03-2026

**To: Editor-in-Chief** (PLOS ONE Journal)

**Subject**: Response to reviewers and revised manuscript **– Epidemiology and Antimicrobial Resistance of Enterobacteriaceae Causing Community-Acquired Urinary Tract Infections in Tétouan, Morocco (2022–2023) (**Manuscript ID: PONE-D-25-62063**)**

**Dear Editor-in-Chief,**

We are pleased to submit the revised version of our manuscript entitled “Epidemiology and Antimicrobial Resistance of Enterobacteriaceae Causing Community-Acquired Urinary Tract Infections in Tétouan, Morocco (2022–2023)” (Manuscript ID: PONE-D-25-62063) for further consideration in PLOS ONE.

We sincerely thank you and the reviewers for your constructive and insightful comments. These suggestions have been invaluable in improving the clarity, rigor, and overall quality of the manuscript.

All reviewer and editor comments have been carefully addressed. A detailed, point-by-point response to each comment is provided, and all revisions have been tracked in the manuscript to ensure transparency.

In addition, the reference list has been thoroughly reviewed to ensure completeness and accuracy. Any retracted articles have been removed or replaced with appropriate, up-to-date references. Where relevant, retained retracted articles have been clearly identified as such in the reference list, with the corresponding retraction notices cited, in accordance with journal recommendations.

**Note:** During final verification of the dataset, we identified that one isolate initially included in the analysis had been misclassified as an Enterobacteriaceae species but was subsequently confirmed as *Sphingomonas paucimobilis*, which does not belong to the Enterobacteriaceae family. To preserve taxonomic accuracy, this isolate was removed from the dataset and all relevant calculations (including total N, percentages, and resistance rates) were recalculated using 422 confirmed Enterobacteriaceae isolates. The revised values differ only minimally from those originally reported and do not alter any of the study’s interpretations or conclusions. We have updated the manuscript accordingly and wish to emphasize that this correction improves the microbiological precision of the work without affecting its scientific integrity or the robustness of the findings.

We hope that the revised manuscript meets your expectations and we look forward to your favorable consideration.

Sincerely,

Ayoub Ez-Zari

**Editor Requirements and comments:**

1. Please ensure that your manuscript meets PLOS ONE's style requirements, including those for file naming. The PLOS ONE style templates.

Response: Thank you for your comment. We confirm that we have revised the manuscript to fully comply with **PLOS ONE** style requirements, including file naming conventions. The manuscript has been formatted according to the official PLOS ONE templates for the main body as well as the title, authors, and affiliations.

2. Please provide additional details regarding participant consent. In the ethics statement in the Methods and online submission information, please ensure that you have specified (1) whether consent was informed and (2) what type you obtained (for instance, written or verbal, and if verbal, how it was documented and witnessed). If your study included minors, state whether you obtained consent from parents or guardians. If the need for consent was waived by the ethics committee, please include this information.

If you are reporting a retrospective study of medical records or archived samples, please ensure that you have discussed whether all data were fully anonymized before you accessed them and/or whether the IRB or ethics committee waived the requirement for informed consent. If patients provided informed written consent to have data from their medical records used in research, please include this information.

Response: We have revised the ethics statement to explicitly clarify the nature of the study, the full anonymization of data prior to access, and the IRB-approved waiver of informed consent, in accordance with the editor’s recommendations.

3. We note that there is identifying data in the Supporting Information file “S1 Supporting information.xlsx”. Due to the inclusion of these potentially identifying data, we have removed this file from your file inventory. Prior to sharing human research participant data, authors should consult with an ethics committee to ensure data are shared in accordance with participant consent and all applicable local laws.

Please remove or anonymize all personal information (<specific identifying information in file to be removed>), ensure that the data shared are in accordance with participant consent, and re-upload a fully anonymized data set. Please note that spreadsheet columns with personal information must be removed and not hidden as all hidden columns will appear in the published file.

Response: We thank the editor for highlighting this important data protection issue. The column entitled **“Code”** in the original S1 Supporting information.xlsx file was generated **after anonymization** of the dataset and was used solely for internal analytical purposes, namely to link biological samples with their corresponding phenotypic and demographic profiles. This code was not derived from, nor linked to, any patient identifiers, medical record numbers, or coordination systems.

Nevertheless, to fully comply with data-sharing and privacy requirements, we have **entirely removed this column**, along with any other variables that could be considered potentially identifying. The Supporting Information file has been revised accordingly and **re-uploaded as a fully anonymized dataset** whit no hiding data.

We confirm that the revised dataset is consistent with the ethical approval granted by the Institutional Review Board and that public data sharing does not compromise participant privacy or confidentiality.

5. Please review your reference list to ensure that it is complete and correct. If you have cited papers that have been retracted, please include the rationale for doing so in the manuscript text, or remove these references and replace them with relevant current references. Any changes to the reference list should be mentioned in the rebuttal letter that accompanies your revised manuscript. If you need to cite a retracted article, indicate the article’s retracted status in the References list and also include a citation and full reference for the retraction notice.

Response: We have carefully reviewed the entire reference list to ensure its accuracy, completeness, and currency. All references have been checked for retraction status. No retracted articles are cited in the revised manuscript. All changes made to the reference list have been incorporated into the revised manuscript.

**Review Comments to the Author:**

**Reviewer #1:**

Farouk et al. described the antimicrobial resistance of bacteria of Enterobacteriaceae family causing urinary tract infections from the hospital/medical laboratories in Tetouan, Morocco. Only two demographic variables gender and age were used to describe antibiotic resistance in bacteria. Further, very limited epidemiological analysis has been done. Many results presented in tables and figures are redundant. The manuscript has limited scope and could be of interest of limited readers.

Response: We thank the reviewer for the constructive comments. Although only age and gender were analyzed, this reflects the nature of routinely available outpatient laboratory data and aligns with the descriptive objective of the study. The primary aim was to provide **baseline, region-specific data on the antimicrobial resistance profiles of community-acquired Enterobacteriaceae in Tétouan** rather than to conduct an exhaustive epidemiological risk factor analysis.

While the scope may appear limited, this study represents the **first such investigation conducted at the provincial level in Tétouan** and forms part of a broader series of studies on infectious diseases in this region. As such, it serves as a **foundational reference** for future, more detailed epidemiological and molecular investigations.

The central contribution of this work is to clarify the **overall community resistance situation**, where empirical antibiotic prescribing is common. These data provide locally generated evidence that can support **updated empirical treatment recommendations and antimicrobial stewardship efforts**.

All tables and figures were reviewed and revised to minimize redundancy and improve clarity.

We believe this study offers valuable regional surveillance data that contribute to the broader understanding of antimicrobial resistance in underreported North African community settings.

1. The short title looks like the key words without any correct meaning. Please correct it.

Response: We appreciate the reviewer’s suggestion regarding the short title. While the original short title was intended to be concise, we agree that it could be improved for clarity and readability. We propose the following alternative short titles: Antibiotic Resistance of Enterobacteriaceae in Community UTI.

2. Although prospective descriptive study has been mentioned in the design, there is no analysis of data as per prospective design. The prospective design has a core aspect of the analysis to track how the measured variables change or evolve over the defined time frame. But there is no such analysis in the manuscript to monitor the trends.

Response: We thank the reviewer for highlighting this point. Upon careful consideration, we acknowledge that our analysis does not track temporal changes in variables over the study period. Although the samples were collected between April 2022 and December 2023, the available data do not allow for time-resolved trend analysis. Therefore, to accurately reflect the study methodology and analysis, we have revised the manuscript to describe the study as a **“cross-sectional descriptive study”** rather than a prospective one. **All instances of “prospective” in the manuscript, including the Abstract, Introduction, and Methods sections, have been updated accordingly.** This modification ensures that the reported study design aligns with the analytical approach presented and maintains full transparency for the readers.

3. ESBL E. coli were 72.7%, which was wrongly calculated. It should be calculated using ESBL E. coli as numerator and total E. coli tested as denominator.

Response: We thank the reviewer for this important observation. We agree that reporting ESBL prevalence within each species provides a more structured and epidemiologically meaningful perspective. In our original manuscript, we reported the predominance of *E. coli* among all ESBL-producing isolates (16/20; 80%), reflecting its overall abundance in the sample. However, as the reviewer correctly points out, the species-specific prevalence (ESBL isolates of a species divided by total isolates of that species) gives a clearer understanding of the relative risk of ESBL production for each species.

In the revised manuscript, we have incorporated both approaches:

1. **Composition of ESBL isolates:** E. coli remains predominant among all ESBL isolates.
2. **Species-specific prevalence:** 4.6% of E. coli and 10.3% of K. pneumoniae isolates were ESBL producers, highlighting the higher likelihood of ESBL production in K. pneumoniae relative to its abundance.

We will also adopt the species-specific prevalence approach in all subsequent comparative analyses, as it provides a more accurate and meaningful assessment of resistance patterns.

4. The resistance trends according to age and gender of the patient have not been discussed. I think these variables although by chance found associated in the results but are not correct variables for comparison.

Response: We thank the reviewer for this valuable comment. Age and sex were included in our analysis as descriptive epidemiological variables, as commonly reported in antimicrobial resistance surveillance studies. In the revised manuscript, we have expanded the Discussion to contextualize the observed age- and sex-related resistance patterns with relevant literature and have explicitly clarified that these associations should be interpreted cautiously. We now emphasize that age and sex were analyzed as descriptive factors rather than causal determinants of antimicrobial resistance, thereby avoiding overinterpretation of these findings.

Discussion part: “Demographic characteristics were associated with distinct antimicrobial resistance patterns in this cohort. Male patients exhibited higher resistance rates to amoxicillin and third-generation cephalosporins, as well as a greater proportion of ESBL-producing isolates, a pattern previously reported in community-acquired urinary tract infections [20,26]. Pediatric isolates showed increased resistance to aminoglycosides and cefotaxime, whereas isolates from elderly patients demonstrated higher resistance to ciprofloxacin and cefoxitin, findings that have been commonly linked in the literature to age-specific antibiotic exposure and patterns of healthcare utilization [20,28,29,33]. These variations underscore the importance of considering demographic characteristics when interpreting local resistance trends; nevertheless, they should be interpreted cautiously, as age and sex were analyzed as descriptive epidemiological factors rather than causal determinants of antimicrobial resistance.”

5. Page 3, line 70, please italicize Klebsiella pneumoniae.

Response: done, and thank you for the remark

6. Page 4, line 104, the prospective design is not appropriate word since there is no such analysis supporting prospective design.

Response: We thank the reviewer for this important observation. Upon careful reconsideration, we agree that the term “prospective” was not appropriate, as the analysis did not include time-resolved or longitudinal assessments. Accordingly, we have revised the manuscript to describe the study as a descriptive cross-sectional study and have implemented all necessary modifications throughout the manuscript to ensure consistency with this study design.

7. Nitrofurantoin is the most commonly used antibiotic for the antibiotic susceptibility test of urine isolates. But it has not been found to be used in this study.

Response: We thank the reviewer for this pertinent observation. Nitrofurantoin is indeed commonly prescribed for urinary tract infections in Morocco and is included in routine urinary antibiograms. However, in our study, it was not displayed in the susceptibility results because our testing and reporting followed standard laboratory protocols, which restrict nitrofurantoin results to specific indications. Specifically: it is generally tested only for cystitis and primarily for *E. coli* isolates; it is not effective against all Enterobacteriaceae (e.g., Proteus spp., *Pseudomonas* spp.); and laboratory interpretative rules may limit its display when other antibiotics provide clearer guidance for therapy. Therefore, its absence in our reported panel reflects laboratory and reporting practices rather than an omission in study design.

8. The distribution of bacterial species according to gender and age has been presented which is not relevant. Similar is the case for distribution of resistance patterns by gender and age. It cannot be explained that pediatric isolates were more likely to harbor resistance to 225 cephalosporins and certain aminoglycosides compared with adult and geriatric isolates.

**Response:** We thank the reviewer for this observation. We acknowledge that age- and gender-specific differences in species distribution and resistance patterns cannot be interpreted as causal and should be considered descriptive only. While we did not emphasize these findings in the discussion, we reported them in the results to provide a complete epidemiological picture of our cohort. We have clarified in the manuscript that these associations are descriptive and should be interpreted cautiously, as they do not imply causation.

9. Table 1 and 2 have presentation of same data. However, there is difference in some data between two tables such as isolates in age group 18-24, 45-64, 65-79 and 80 or more.

**Response:** We thank the reviewer for pointing out the redundancy between Tables 1 and 2, as well as the discrepancies in some age groups. After careful review, we agree that presenting both tables separately was unnecessary. The discrepancies in the first table were due to the use of an unupdated version of the crude data.

To address this, we have **merged the descriptive sociodemographic information and the univariate logistic regression analysis into a single table** (now Table 1). This table includes both the frequencies and percentages of patients by age and gender, as well as the corresponding odds ratios and confidence intervals from the univariate analysis. The new title—“Sociodemographic characteristics and univariate logistic regression of gender distribution across age groups among urinary tract infection patients in Tétouan, Morocco, 2022–2023 (N=422)”—reflects this combined purpose.

This revision **eliminates redundancy, corrects the earlier data inconsistencies, and provides a comprehensive, clear, and reviewer-proof presentation of the demographic findings.**

10. Table 3 and figure 1, and table 4 and figure 2, present the same data. Therefore, one is redundant.

We thank the reviewer for this observation. We agree that Table 3 and Figure 1, as well as Table 4 and Figure 2, presents redundant data. To avoid redundancy while maintaining clarity, we have **retained the tables**—, which provide precise numerical values—and **removed the corresponding figures**. Although figures can visually enhance the results, we believe that the tables alone sufficiently present the data without duplication, ensuring a concise and clear presentation.

11. As stated by authors, there are several limitations of the study. There are very limited data described based on very few variables.

Response: We thank the reviewer for highlighting this important point. We fully acknowledge that the study is based on a limited number of variables, which restricts the depth of epidemiological and resistance analyses. This limitation is inherent to the available dataset and the scope of our laboratory records.

Nevertheless, the study provides a **public health-oriented overview** of urinary tract infection pathogens and resistance patterns, offering clinicians a practical foundation for empirical prescribing decisions in the local context. By systematically presenting antimicrobial susceptibility, ESBL production, and demographic distributions, our work aims to guide evidence-based treatment choices and inform future, more detailed studies, even with the limited variables available.

**Reviewer #2:**

The authors present a well-conducted prospective study addressing an important public health issue. The methodology is sound, and the findings provide valuable local data on antimicrobial resistance patterns, especially for the northern regions of Morocco. However, minor revisions are suggested to improve clarity, accuracy, and reader-friendliness.

Specific Comments:

1) In lines 61–62, the authors should consider citing more relevant and up-to-date references to strengthen the context.

Response:
We thank the reviewer for this helpful suggestion. In response, we have added a recent and comprehensive review to strengthen the contextual background on UTIs: Mancuso G, Midiri A, Gerace E, Marra M, Zummo S, Biondo C. Urinary tract infections: the current scenario and future prospects. Pathogens. 2023;12(4):623. This reference has been incorporated in lines 61–62 of the Introduction to provide a more up-to-date overview of the current epidemiological and clinical landscape of UTIs.

2) Please ensure that abbreviations for binomial names, such as E. coli, are italicized throughout the manuscript. For examples, lines 171–172 require correction, also in line 70. Please check the entire text for consistency.

Reponses: Thank you for pointing this out. We have ensured that all species names are now italicized consistently throughout the manuscript.

3)The methods are generally well described. However, the following points require attention. The phrase "in several medical laboratories" is vague. Please specify the number and type of laboratories (e.g., hospital-based, private, etc.) to enhance reproducibility. Moreover, specify whether consecutive or random sampling was used for isolate collection.

Response: We thank the reviewer for this comment. The study was conducted in the three largest private medical laboratories located in the city center of Tétouan, and urine specimens were collected consecutively throughout the study period. The manuscript has been updated to include these details to enhance reproducibility.

4) Please clarify that samples were only first episodes included, or were recurrent UTIs included? Were symptomatic and asymptomatic bacteriuria both included?

Response: We thank the reviewer for this comment. All isolates came from different patients, so each represents a single episode of bacteriuria. Unfortunately, no clinical records were available, and therefore we cannot distinguish between first or recurrent UTIs, nor between symptomatic and asymptomatic bacteriuria. This limitation has now been clarified in the Methods section (included in Inclusion and exclusion criteria).

5) For consistency in data presentation, ensure all percentages throughout the manuscript (including the abstract) use the same number of decimal places. As an example, 4.97% could be rounded to 5.0% to match 74.7%, or both could be presented with two decimals (74.70% and 4.97%).

Response: We thank the reviewer for this suggestion. All percentages throughout the manuscript, including the abstract, have been standardized to one decimal for consistency.

6) The presentation of Figures 1, 2, and 3 could be enhanced. I recommend improving the resolution, labels, and overall clarity to make the data more easily interpretable.

Response: We thank the reviewer for this suggestion. To avoid redundancy, Figures 1 and 2 have been removed, as the data are already fully presented in the tables. Figure 3 has been retained and its resolution, labels, and overall clarity have been refined and enhanced for better interpretability.

7) Lines 244–245 require revision to ensure accuracy. Please rephrase accordingly.

Response: We thank the reviewer for this comment. Lines 244–245 have been revised for clarity and accuracy as follows: “Urinary tract infections (UTIs) are among the most common bacterial infections globally, with Enterobacteriaceae being the predominant causative agents [13].”

8)Line 266: Full spelling of Klebsiella pneumoniae is unnecessary as the abbreviation has already been introduced. Please revise and ensure scientific writing quality throughout.

Response: We thank the reviewer for this comment. The full spelling of Klebsiella pneumoniae in line 266 has been replaced with the previously introduced abbreviation K. pneumoniae. We have also carefully reviewed the manuscript to ensure consistent and high-quality scientific writing.

9) The conclusion would be strengthened by briefly acknowledging study limitations (e.g., single region, short duration) to avoid overgeneralization.

Response: We thank the reviewer for this suggestion. The Conclusion has been revised to briefly acknowledge the study’s limitations, including its restriction to a single region and study period.
